# In vitro investigation of the effect of proinflammatory cytokines on mouse choroid plexus membrane transporters Ncbe and NKCC1

**DOI:** 10.1186/s12987-023-00474-9

**Published:** 2023-10-12

**Authors:** Laura Øllegaard Johnsen, Kathrine Abildskov Friis, Helle Hasager Damkier

**Affiliations:** Department of Biomedicine, Faculty of Health, Aarhus University, Aarhus, Denmark

**Keywords:** Cerebrospinal fluid, Intraventricular hemorrhage, Post-hemorrhagic hydrocephalus

## Abstract

Intraventricular hemorrhage is a potentially life-threatening condition. Approximately 20% of patients develop posthemorrhagic hydrocephalus with increased ventricular volume and intracranial pressure. Hydrocephalus develops partially due to increased secretion of cerebrospinal fluid by the choroid plexus. During hemorrhage a multitude of factors are released into the cerebrospinal fluid. Many of these have been implicated in the hypersecretion. In this study, we have investigated the isolated effect of inflammatory components, on the abundance of two membrane transporters involved in cerebrospinal fluid secretion by the choroid plexus: the Na^+^-dependent Cl^−^/HCO_3_^−^ exchanger, Ncbe, and the Na^+^, K^+^, 2Cl^−^ cotransporter, NKCC1. We have established a primary choroid plexus epithelial cell culture from 1 to 7 days old mouse pups. Seven days after seeding, the cells formed a monolayer. The cells were treated with either tumor necrosis factor alpha (TNFα), interleukin 1 beta (IL-1β), or interleukin 6 (IL-6) to mimic inflammation. The data show that treatment with TNFα, and IL-1β only transiently increased NKCC1 abundance whereas the effect on Ncbe abundance was a transient decrease. IL-6 however significantly increased NKCC1 (242%), the phosphorylated NKCC1 (147%), as well as pSPAK (406%) abundance, but had no effect on Ncbe. This study suggests that the inflammatory pathway involved in hypersecretion primarily is mediated by activation of basolateral receptors in the choroid plexus, mainly facilitated by IL-6. This study highlights the complexity of the pathophysiological circumstances occurring during intraventricular hemorrhage.

## Background

A subarachnoid hemorrhage (SAH) is most often the result of a rupture of one of the major arteries of the Circle of Willis on the base of the brain, and is caused by leakage of an aneurism [1]. The rupture leads to large amounts of blood pulsating into the subarachnoid space. In severe cases, SAH leads to an intraventricular hemorrhage (IVH) as blood flows from the subarachnoid space into the brain ventricles. The presence of blood in the brain ventricles has a negative impact on survival [2]. Approximately 20–30% of patients with SAH develop post-hemorrhagic hydrocephalus (PHH), with the possibility of neurological impairments and death [3]. Hydrocephalus is generally defined as a disorder of cerebrospinal fluid (CSF) physiology that results in expansion of the cerebral ventricles and is in some cases followed by increased intracranial pressure [4]. PHH may partly be explained by mechanical obstruction or decreased reabsorption in the arachnoid granulations that absorb the majority of CSF but is generally believed to be multifactorial [3]. One factor following SAH is increased secretion of CSF by the choroid plexus (CP) [5, 6]. A number of factors have been implicated in the increased CSF secretion including release of proinflammatory cytokines [6, 7] and hemolysis with release of hemoglobin and iron [8, 9]. The increase in CSF secretion and development of PHH occurs acutely within the first 1–2 days following the onset of hemorrhage. While some cases of hydrocephalus do persist as a chronic condition most hydrocephalus cases following hemorrhage are transient and cleared within a few days to weeks [10].

The CP is an actively secreting epithelial structure located in the brain ventricles. It forms the blood-CSF barrier (BCSFB) and secretes approximately 80% of the CSF [11]. The secretion rate of the CP is 0.4 mL/min and, with a total volume of CSF estimated to half a liter, the CP replaces the CSF volume three times a day in humans [12]. Thus, alterations in the CSF flow, reabsorption, or secretion, can rapidly lead to an increased intracranial pressure and hydrocephalus because of the rigidity of the skull [13].

CSF secretion is believed to be driven by the luminally located Na^+^/K^+^ ATPase, by its direct extrusion of Na^+^ into the CSF [14–17]. The Na^+^, K^+^, 2Cl^−^ cotransporter, NKCC1 is also located in the luminal membrane [18, 19]. The luminal position, and the bumetanide sensitivity towards CSF secretion, shows that NKCC1 plays a role in CSF secretion [20, 21]. Whether the role of NKCC1 is to directly extrude Na^+^ from CP to CSF or to import Na^+^ and K^+^ from CSF to drive the extrusion of Na^+^ by the Na^+^/K^+^ ATPase while removing K^+^ from CSF is a subject of debate [22–24].

The Na^+^ dependent Cl^−^/HCO_3_^−^ exchanger, Ncbe, is located in the basolateral membrane of the CP epithelial cells where it is believed to be the key basolateral Na^+^-loader involved in CSF secretion [12]. Mice with genetic ablation of Ncbe present with smaller brain ventricles [25] indicating that Ncbe is vital for CSF secretion. A study in neonatal rats showed increased expression of Ncbe following intracranial hemorrhage leading to increased CSF production [26], which could be hindered by genetically deleting Ncbe.

Following an IVH a multitude of factors are released into the CSF. One of these factors is a range of cytokines [27]. The two most prevalent and most abundant cytokines in CSF from SAH patients are the pro-inflammatory cytokines TNFα and IL-1β [7]. In addition to these two, most studies also find an increased abundance of IL-6 [7]. TNFα and IL-1β both act on receptors found in the luminal membrane of the CPE cells, similar to TLR4 [28, 29]. TNFα binds to the TNFR and IL-1β binds to the IL-1 receptor. The TLR4, TNFR, as well as the IL-1 receptor signal through the NFkB pathway. IL-6, on the other hand, exerts its function on the IL-6Rα which is predominantly expressed on the basolateral side of the CPE cells [29].

As mentioned earlier, previous studies found that hemorrhage-induced inflammation leads to increased CSF secretion [6, 30]. Usually, inflammation is a protective response which helps eliminate the cause of injury, initiate tissue repair, and clear out resulting debris. Increased secretion of CSF could be a mechanism for the epithelial cells to clear the inflammatory mediator present in the luminal space.

Many other factors are released during IVH that could affect expression of the transporters in the CP involved in CSF secretion. To investigate each factor individually and to avoid the hemodynamic factors, we aimed to establish an in vitro model of the BCSFB and in this study to isolate the effects of selected proinflammatory cytokines. We investigated the abundance of two important transporters involved in CSF secretion: the luminal NKCC1 and the basolateral Ncbe.

In this study, we show that TNFα, and IL-1β, that affect CP luminal receptors, transiently decrease abundance of Ncbe in the CP. IL-6 that acts on the basolateral receptor, does not affect Ncbe abundance. NKCC1, on the other hand, is primarily affected by IL-6, which increases the abundance of both NKCC1 and the active form pNKCC1. The inflammatory response mediated by the most common pro-inflammatory cytokines of IVH (TNFα and IL-1β) indicate a polarized effect on the membrane transporters. This suggests that the inflammatory response is not only mediated by a direct effect of cytokines released into CSF but also via basolateral pathways.

## Methods

### Experimental animals

C57BL/6J mouse pups between postnatal day 1 and 7 (P1-P7) were used to establish primary choroid plexus epithelial (CPE) cell cultures. All experiments were performed within the current guidelines of the Danish Animal Research Inspectorate and ethical care.

### Establishment of the choroid plexus primary monoculture

Primary cell cultures of mouse CPE cells were established according to a modified protocol from Menheniott et al. [31].

Mouse pups (P1-P7) were decapitated, and the brains were isolated. The CP from the lateral and fourth ventricles were dissected and isolated in ice-cold 0.1 M PBS (NaCl: 137mM, KCl: 2.7 mM, Na_2_HPO_4_: 10 mM, KH_2_PO_4_: 1.8 mM) and then transferred to basal culture medium (DMEM-F12 (BioWest, #L0092-500) + 10% FBS (Gibco, #10270-106) + 1% P/S (BioWest, #L0022-100)) and placed on ice. The tissue was kept on ice until further processing. The tissue was washed in 37 °C warm PBS and dissociated enzymatically by incubation with 1 mg/mL pronase (isolated from *Streptomyces griseus*, Roche, #10-165-921) for 25 min. Digestion was stopped by centrifugation of the tubes and removing the enzymatic solution. The pellet was resuspended and washed in 37 °C warm PBS. Next, the cells were briefly incubated with 0.025% trypsin (BioWest, #L0930-500) and 6.25 µg/mL DNase I (Roche, #10-104-159) in PBS, followed by gentle trituration using a P1000 pipette. Digestion was stopped by addition of basal culture medium to the enzymatic solution. The cells were spun down, and the pellet was resuspended in 37 °C growth medium (basal culture medium supplemented with 0.1x Insulin-Transferrin-Selenium (Gibco, #41400-045), 2mM L-glutamine (Gibco, #25030-024), 20 ng/mL epidermal growth factor (Sigma, #62229-50-9), and 5 ng/mL basic fibroblast growth factor (Sigma, #3685)) and plated on collagen IV (Sigma, #C5533) coated filter inserts in a 24-well plate (Nunc™ Polycarbonate Cell Culture Inserts [140,620]). The following day, fresh growth medium supplemented with 30 µM cytosine arabinoside (Ara-C, Sigma, #147-94-4) was added to the culture to eliminate the growth of fibroblasts. Cultures were maintained at 37 °C in 5% CO_2_ during the growth period and fresh growth medium was added every other day. Ara-C supplement was included in the first two media changes. Seven days after culture establishment, the culture had reached confluency and experimental treatment was initiated.

### Transepithelial electrical resistance (TEER) measurements

TEER values were measured using chopstick electrodes connected to an Epithelial Volt/Ohm Meter (EVOM™). Each TEER value is based on an average of three measurements distributed evenly within the insert. TEER values were calculated by:

$$TEER=\left({R}_{Total}-{R}_{Blank}\left[{\Omega }\right]\right)\times {M}_{Area}\left[{cm}^{2}\right]$$

Where ${R}_{Total}$ (Ω) is the total resistance (average of three measurements) measured in the insert and ${R}_{Blank}$ (Ω) is the resistance measured in an empty filter conditioned with cell growth medium (180 Ω). ${M}_{Area}$ (cm^2^) is the effective area of the semipermeable membrane which for the Nunc™ Polycarbonate Cell Culture Inserts (ThermoFisher Scientific, #140,620) is 0.47 cm^2^.

### Treatment of CPE cells with proinflammatory cytokines

Imitation of IVH inflammation was introduced by treating the cells with 1 pg/mL, 1ng/mL or 100ng/mL TNF-α (Sigma, #T7539), IL-1β (Sigma, #SRP8033), or IL-6 (Sigma, #SRP3330). The inflammatory factors were introduced to the cells in the treatment medium in both the upper and lower chamber to ensure activation of the receptors irrespective of membrane localization. The cells were cultured in 5% CO_2_ and at 37 °C and for the 72-hour experiments, new treatment medium supplemented with the inflammatory factor was added after 48 h.

### Immunoblotting

The cells were harvested by dissolving in sample buffer (1.5% (w/vol)) SDS, 40.0 mm 1,4-dithiothreitol, 6% (v/v) glycerol, 10 mm Tris(hydroxymethyl)-aminomethane, pH 6.8, and bromophenol blue) with a 7.5 mg/mL DTT supplement. After harvesting, the samples were boiled at 95 °C for five minutes followed by a thorough vortex and sonication for cell lysis and stabilization of the proteins. Samples were sonicated by five pulses at 20% using a Model 150 V/T sonicator (BioLogics Inc., Cary, NC, USA).

To minimize variation in total protein load, a Coomassie assay was performed. Proteins were separated by Sodium dodecyl sulfate-polyacrylamide gel electrophoresis (SDS PAGE) in a 4–15% precast polyacrylamide gel (Bio-Rad, #5,671,085) along with a protein marker (Bio-Rad, #1,610,373). The gel was run at a constant 200 V and stained with Gel Code (Thermo Fisher, #24,590) for 1 h. The gel was destained in MiliQ water over night at 4 °C. The protein bands were developed using the LI-COR Odyssey 1.2 platform.

The proteins were transferred to a PVDF membrane using the Trans-Blot turbo system (BioRad #1,704,273). The transfer was run at 25 V and up to 1.0 A for 30 min. After transfer the membrane was blocked in 5% skimmed milk in PBS-T or 5% BSA in PBS-T (pNKCC1 blots) to block nonspecific protein binding. The membrane was incubated with primary antibodies diluted in primary antibody diluent (2% BSA, 2 µM NaN_3_, 0.1% Tween in 0.1 M PBS) over night at 4 °C. After incubation, the membrane was washed in PBS-T and incubated with appropriate HRP-conjugated secondary antibodies (Invitrogen) in 5% skim milk for one hour at room temperature. The membrane was developed using ECL western blotting detection reagent (Sigma, #GERPN3243). The blots were imaged using the Invitrogen iBright 1500 and densitometric analysis was performed using ImageJ.

### Immunocytochemistry

CPE cells grown on filter inserts or coverslips were fixed in 4% paraformaldehyde (PFA) in PBS. After fixation, the cells were permeabilized using a permeabilization buffer (0.2% saponin in 100 mL 0.1 M PBS). The cells were then blocked with two blocking solutions: 1 (10% FBS, 0.1% BSA, 0.05% Saponin in 0.1 M PBS) and 2 (1% BSA, 0.2% Fish gelatine, 0.05% Saponin, 0.3754% Glycine in 0.1 M PBS). The cells were then incubated over night at 4 °C with primary antibodies. Antibodies used in this experiment are listed in Table 1. The fluorescence visualization of the primary antibodies was performed using AlexaFlour 488- or 555-coupled secondary antibodies (Invitrogen). After incubation, the cell nuclei were counterstained with To-Pro (Invitrogen). After the final stain, the cells were washed and mounted using fluorescent mounting medium (Dako, # S3023). The cells were imaged using a Leica DMIRE2 inverted microscope with a TC5 SPZ confocal unit using a 63×/1.32 NA objective. Image stacks were made using a z-distance of 0.15 μm between images. Images were visualized using ImageJ software.

**Table 1:** List of antibodies

Target	Antibody no.	Host	Source
Ncbe	1139AP	Rabbit	[32]
pNKCC1	Abs1004	Rabbit	Sigma
NKCC1	Ab59791	Rabbit	Abcam
pSPAK	07-2273	Rabbit	Milipore
Proteasome 20 S (H-120)	sc-67,339	Rabbit	Santa Cruz
Na^+^/K^+^-ATPase β1	MA3-930	Mouse	Thermo Scientific
Claudin 2	SAB4503544	Rabbit	Sigma
Occludin	SAB4200593	Rabbit	Sigma

### Statistical analysis

TEER control values over time were analyzed by one-way ANOVA followed by multiple comparisons analysis comparing TEER-values to day 3 after seeding to evaluate the development of the primary culture. TEER values were analyzed by two-way ANOVA followed by multiple comparisons analysis comparing the cultures exposed to different treatment factors to time-matched control cultures as well as the effect of treatment within each treatment group. Protein abundance was analyzed by one-way ANOVA followed by multiple comparisons analysis, similarly, comparing cultures exposed to different treatment factors to control cultures. The results are shown as mean ± standard error of the mean (SEM). The *n* denotes a CPE cell culture from one mouse pup.

## Results

### Choroid plexus epithelial cells in culture establish a monolayer with tight junctions and correctly polarized membrane transporters

**Fig. 1:**
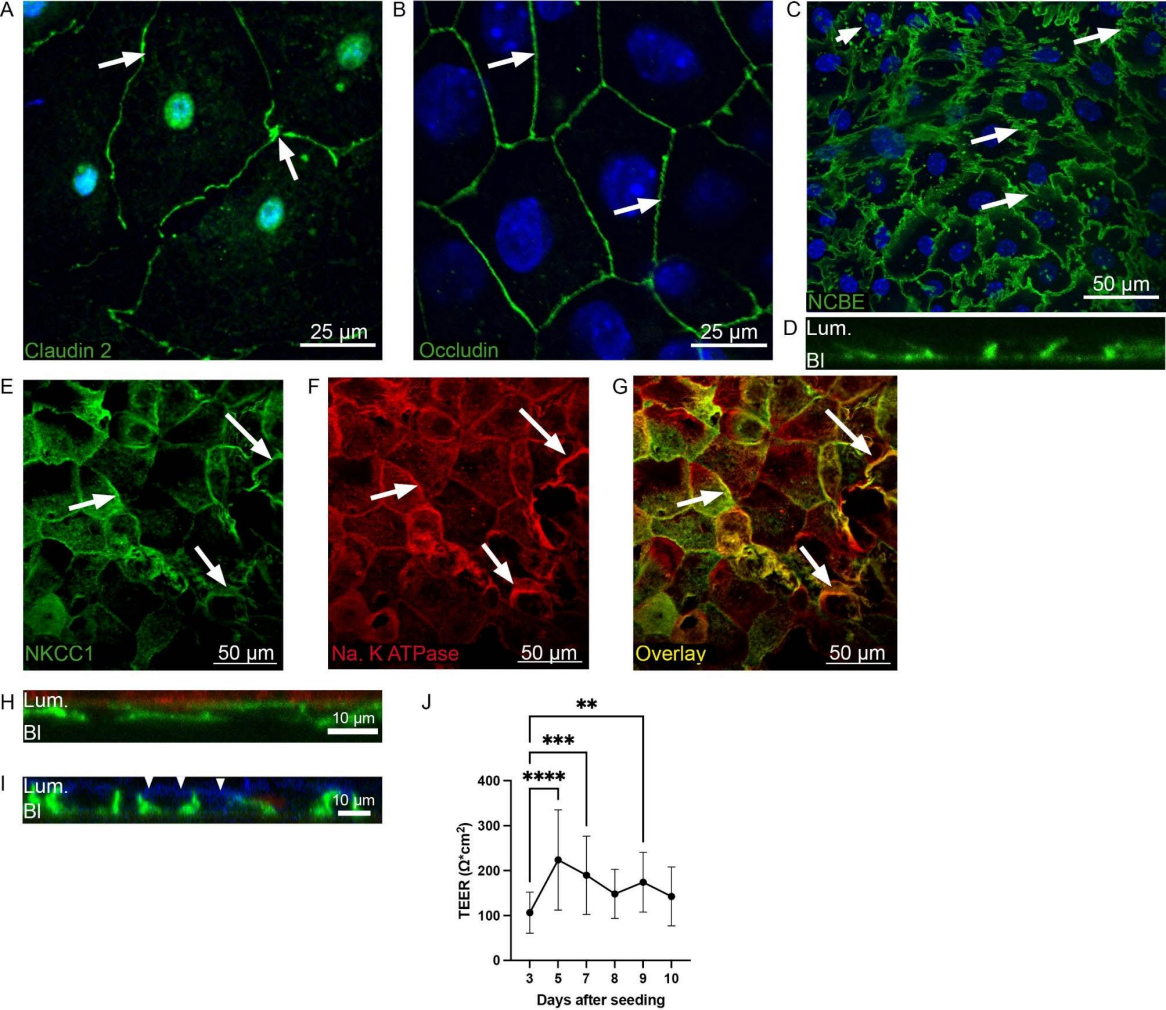
**Expression of cell-cell contact proteins and membrane transporters in the mouse choroid plexus primary culture**. Immunofluorescence labeling of mouse choroid plexus primary culture for the tight junction proteins Claudin 2 (**A**) and Occludin (**B**). Arrows indicate intercellular junctions expressing both proteins. Nuclei (blue) are stained with Topro. **C**) Immunofluorescence labeling of cultures for the Na^+^ dependent Cl^−^/HCO_3_^−^ exchanger, Ncbe (green), in the basolateral membrane. The arrows indicate examples of the basolateral labyrinths; characteristic of the CP. Ncbe-specific labelling is also seen in intracellular vesicles (arrow heads). Cell nuclei are stained with Topro nuclear stain (blue). A z-stack through the cells confirms basolateral Ncbe (green) expression (**D**). Immunofluorescense labelling confirms expression of NKCC1 (green, **E**) and Na, K ATPase (Red, **F**) in the membrane of the CP cells. Overlaying the images, the yellow color confirms colocalization of the two luminal transporters (**G**). Arrows indicate examples of the membrane specific staining (**E** and **F**) as well as subcellular co-localization in the overlayed image (**G**). Z-stacks confirm correct polarization of the luminal Na, K ATPase (red, **H**) and NKCC1 (blue color at arrow heads, **I**) when co-labelled with Ncbe (green). (**J**) Trans-epithelial electrical resistance measured at 3, 5, 7, 8, 9 and 10 days after seeding. TEER values represent mean ± SEM. **indicates p < 0.01 and **** indicates p < 0.0001

The CPE cells grew to confluency within a week after establishing the culture and showed the typical cobblestone morphology (Fig. 1A-C). The cells showed positive staining for the tight junction proteins Claudin 2 (Fig. 1A) and Occludin (Fig. 1B). When grown on polycarbonate filters, the morphology of the CPE cells closer resembled CP morphology, showing basolateral infoldings that are characteristic for the CP epithelial cell layer (Fig. 1C).

Confocal microscopy confirmed proper polarization of three transporters in the CP. Ncbe is expressed in the basolateral membrane (Fig. 1C and D). The cells expressed the Na^+^, K^+^, 2Cl^−^ transporter, NKCC1 (Fig. 1E) and the Na^+^/K^+^ ATPase (Fig. 1F). Overlaying the images, confirmed that the two luminal proteins were found in the same part of the plasma membrane. Double labelling using Ncbe as a basolateral marker with either Na^+^/K^+^ ATPase (Fig. 1H) or NKCC1 (Fig. 1I) specific antibodies confirmed the correct luminal plasma membrane localization of the two transporters.

Confluency and integrity of the cell culture was evaluated by measuring the TEER values (Fig. 1J). TEER values in control cultures significantly increased from 107 ± 8 Ω*cm^2^ at day 3 (n = 32) to 224 ± 20 Ω*cm^2^ at day 5 (n = 32) and 190 ± 16 Ω*cm^2^ at day 7 (n = 29, TEER day 3 versus day 5: p < 0.0001; day 3 versus day 7: p = 0.0002). In the control cells, TEER levels plateaued at 148 ± 17 Ω*cm^2^ at day 8 (n = 10), 174 ± 15 Ω*cm^2^ at day 9 (n = 19) and 143 ± 20 Ω*cm^2^ at day 10 (n = 11). Treatment was initiated after one week, when the cells exhibited the highest TEER values. The TEER values were also evaluated during and at the end of the treatment regimens. No significant differences were found in TEER values when comparing the end TEER value in the control group with each of the treatment groups (Table 2). When comparing the TEER value at the start of the experiment with the value at the end in each treatment group, no differences were observed at lower doses of the cytokines (1 pg/mL and 1 ng/mL, Table 2). A statistically significant difference was, however, observed when comparing the TEER value after 24 h treatment with 100 ng/mL TNFα and IL-1β, respectively. In both groups, there was a significant decrease in TEER after treatment (Table 2). When comparing the TEER values in the 48-hour experiments, however, the difference was not detected. No difference in TEER was observed with IL-6 treatment in either the 24- or 48-hour experiments, but after 72 h treatment with 100 ng/mL there was a significant decrease in TEER in the IL-6 treated cells. These experiments suggest that higher concentrations of TNFα and IL-1β possibly can cause a transient disruption of the barrier between the CP cells. IL-6, however, does not seem to disrupt the barrier in the first two days, but prolonged exposure after 72 h can possibly affect the barrier-function of the CP.

**Table 2:** Values are presented as mean ± standard error; n.s.:no statistical significance, and *p < 0.05, n = 5

TEER values (Ω*cm^2^)					
TNFα					
	Treatment	Control	1 pg/mL	1 ng/mL	100 ng/mL
24 h	Start	138 ± 8	149 ± 8	154 ± 11	157 ± 8
	End	122 ± 8 ^n.s^.	140 ± 11 ^n.s^.	132 ± 6 ^n.s^.	132 ± 7*
48 h	Start	217 ± 12	195 ± 14	212 ± 20	230 ± 22
	End	233 ± 22 ^n.s^.	218 ± 3 ^n.s^.	229 ± 37 ^n.s^.	206 ± 10 ^n.s^.
IL-1β					
	Treatment	Control	1 pg/mL	1 ng/mL	100 ng/mL
24 h	Start	143 ± 13	140 ± 14	144 ± 12	165 ± 13
	End	145 ± 19 ^n.s^.	157 ± 18 ^n.s^.	131 ± 19 ^n.s^.	132 ± 15*
48 h	Start	143 ± 9	136 ± 9	135 ± 7	150 ± 7
	End	142 ± 19 ^n.s^.	136 ± 18 ^n.s^.	145 ± 13 ^n.s^.	165 ± 18 ^n.s^.
IL-6					
	Treatment	Control	1 pg/ml	1 ng/ml	100 ng/ml
24 h	Start	132 ± 4	127 ± 9	137 ± 4	143 ± 7
	End	141 ± 7 ^n.s^.	138 ± 5 ^n.s^.	139 ± 12 ^n.s^.	147 ± 6 ^n.s^.
48 h	Start	174 ± 18	195 ± 19	164 ± 15	178 ± 29
	End	190 ± 15 ^n.s^.	204 ± 16 ^n.s^.	193 ± 12 ^n.s^.	190 ± 27 ^n.s^.
100 ng/mL prolonged treatment					
	Treatment	Control	TNFα	IL-1β	IL-6
72 h	Start	170 ± 30	177 ± 54	152 ± 30	221 ± 20
	End	147 ± 27 ^n.s^.	148 ± 29 ^n.s^.	159 ± 21 ^n.s^.	166 ± 14*

### Acute treatment of the CPE cultures with the proinflammatory cytokines TNFα and IL-1β transiently reduces abundance of Ncbe, whereas the abundance of Ncbe is unaffected by IL-6

To investigate the effect of each of the most prominent proinflammatory cytokines during IVH, cells were treated with either TNFα, IL-1β, or IL-6 at three different concentrations for either 24 or 48 h and Ncbe abundance was compared to control levels. TNFα and IL-1β both act through the NFΚB pathway mediated through the TNFR, TLR or IL-1R expressed in the luminal membrane of the CP. IL-6 acts through the JAK/STAT pathway and the IL-6 receptor is expressed in the basolateral membrane of the CP.

**Fig. 2:**
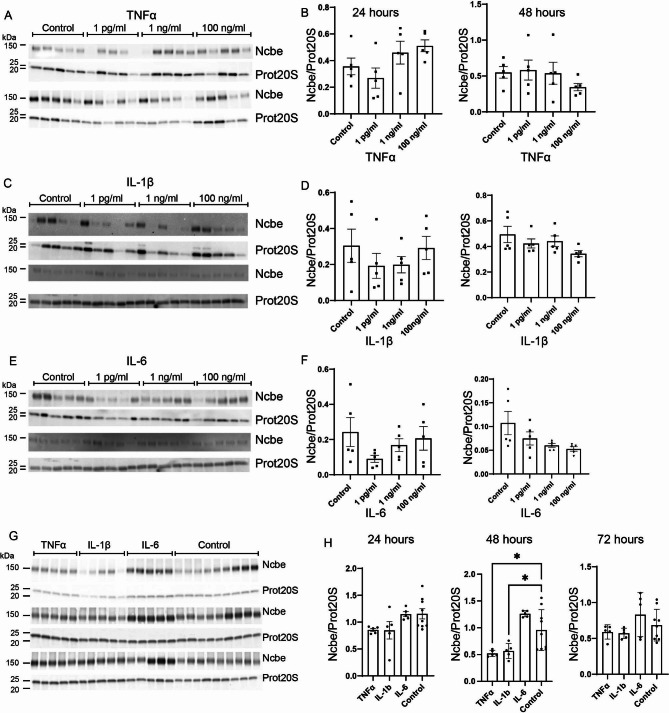
**Effect of proinflammatory cytokines TNFα, IL-1β and IL-6 on CP expression of the basolateral transporter, Ncbe**. Primary cultures were treated with 1 pg/mL, 1 ng/mL, or 100 ng/mL of either TNFα (**A** and **B**), IL-1β (**C** and **D**) or IL-6 (**E** and **F**) for 24 and 48 h. (**G** and **H**). Treatment with 100 ng/mL TNFα, IL-1β and IL-6 for 24, 48 and 72 h. Cells were lysed and subjected to immunoblotting for Ncbe and proteasome 20 S on the same membrane. Top two bands in A, C, E and G represent Ncbe and prot20S after 24 h treatment and lower two bands represent Ncbe and prot20S after 48 h treatment. Molecular masses (shown in kDa) are indicated on the left. The Ncbe and proteasome 20 S specific bands were quantified densiometrically. Bargraphs in B, D, F, and H show the Ncbe abundance relative to proteasome 20 S as mean ± SEM. *indicates p < 0.05

Treatment for 24 h with TNFα (Fig. 2A top and B left) did not change Ncbe abundance compared to control using either 1 pg/mL, 1 ng/mL, or 100 ng/mL. Treatment for 48 h with TNFα (Fig. 2A bottom and 2B right) also did not change Ncbe abundance at either 1 pg/mL, 1 ng/mL, or 100 ng/mL.

Furthermore, 24 h treatment with IL-1β (Fig. 2C and D) did not significantly change Ncbe abundance at either 1 pg/mL, 1 ng/mL, or 100 ng/mL or after 48 h at 1 pg/mL, 1 ng/mL, or 100 ng/mL.

Finally, treatment with IL-6 did not significantly alter Ncbe abundance after 24 h (Fig. 2E and F) using either 1 pg/mL, 1 ng/mL, or 100 ng/mL, or after 48 h at 1 pg/mL, 1 ng/mL, or 100 ng/mL.

While there was no significant effect on Ncbe abundance using either of the cytokines, there seemed to be a tendency to a decreased abundance of the transporter after the longest exposure time (48 h) using the highest concentration of cytokines (100 ng/mL). Furthermore, TEER values suggested that at least TNFα and IL-1β at the highest concentration could transiently cause a disruption of the barrier. To investigate whether prolonged exposure to cytokines would affect Ncbe abundance, the experiments were repeated investigating the effect of the highest dose (100 ng/mL) but at three time points.

In the repeated experiments, protein abundance of Ncbe remained unchanged following 24-hour treatment (Fig. 2G top and H left) with either TNFα, IL-1β, or IL-6. In these set of experiments, however, both TNFα and IL-1β reduced abundance of Ncbe by 45% (95% CI of diff. [0.075 to 0.80], p = 0.0158) and 41% (95% CI of diff. [0.03 to 0.75], p = 0.0316), respectively (Fig. 2G middle and 2 H middle) after 48 h, while Ncbe remained unaffected by treatment with IL-6.

After 72 h, Ncbe abundance was restored to normal in the TNFα, IL-1β, and IL-6-treated cell culture (Fig. 2G bottom and 2 H right).

These latter results suggest that the regulation of Ncbe by inflammation is mediated by the luminally placed receptors as both TNFα and IL-1β treatment transiently decreased abundance of Ncbe. The difference in the two sets of experiments could indicate that the timeframe in which Ncbe is reduced is very short and transient.

### Acute treatment of the CPE cultures with TNFα reduces pNKCC1 but does not affect pSPAK and total NKCC1

**Fig. 3:**
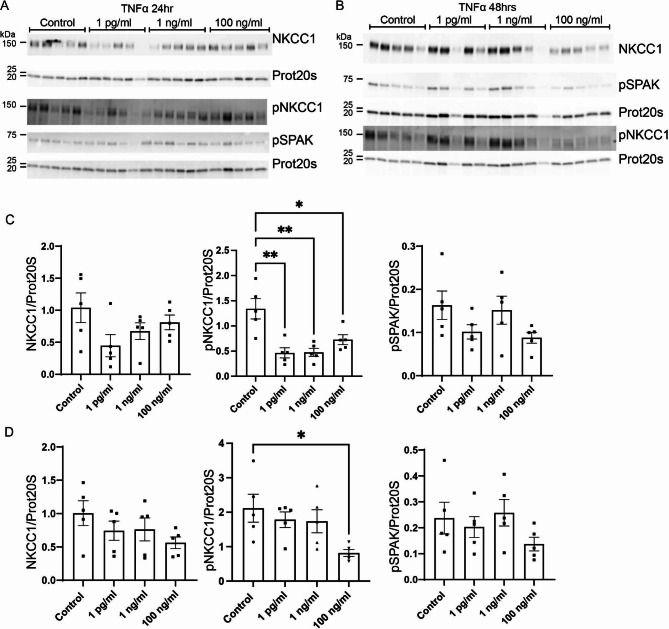
**Effect of the proinflammatory cytokine TNFα on the CP expression of NKCC1, pNKCC1 and pSPAK.** Primary cultures were treated with either 1 pg/mL, 1 ng/mL, or 100 ng/mL TNFα for 24 (**A**) or 48 (**B**) hours. Cells were lysed and subjected to immunoblotting for NKCC1, pNKCC1, pSPAK and proteasome 20 S on the same membrane. Molecular masses (shown in kDa) are indicated on the left. The NKCC1, pNKCC1, pSPAK and proteasome 20 S specific bands were quantified densiometrically. Bargraphs in (**C**) and (**D**) show the ratio of NKCC1/proteasome 20 S (left), pNKCC1/proteasome 20 S (middle), and pSPAK/proteasome 20 S (right) as mean ± SEM. *indicates p < 0.05 and **p < 0.01

Previous publications have shown that the inflammatory response following IVH leads to activation of NKCC1 via the TLR4 receptor. The active form of NKCC1 is the phosphorylated form of NKCC1, pNKCC1, and NKCC1 is normally phosphorylated by the WNK/SPAK pathway. The 24-hour treatment of the cells with TNFα (Fig. 3A and C) did not affect abundance of total NKCC1 at either 1 pg/mL, 1 ng/mL, or 100 ng/. The activated form of NKCC1, pNKCC1, was however significantly reduced by 65% with 1 pg/mL TNFα (95% CI of diff. [0.35 to 1.40], p = 0.001), by 64% with 1 ng/mL (95% CI of diff. [0.34 to 1.39], p = 0.0012), and by 46% with 100 ng/mL TNFα (95% CI of diff. [0.09 to 1.14], p = 0.019). The treatment did, however, not change abundance of pSPAK after 24 h of treatment with TNFα. Treatment with TNFα for 48 h (Fig. 3B and D) similarly did not affect total NKCC1 either at 1 pg/mL, 1 ng/mL, or 100 ng/mL. The phosphorylated and active form of NKCC1 remained reduced by 61% (95% CI of diff. [0.12 to 2.48], p = 0.028) but only with 100 ng/mL TNFα, whereas the lower doses had no effect. Similar to 24 h of treatment with TNFα, treatment for 48 h did not affect pSPAK at either 1 pg/mL, 1 ng/mL, or 100 ng/mL.

**Fig. 4:**
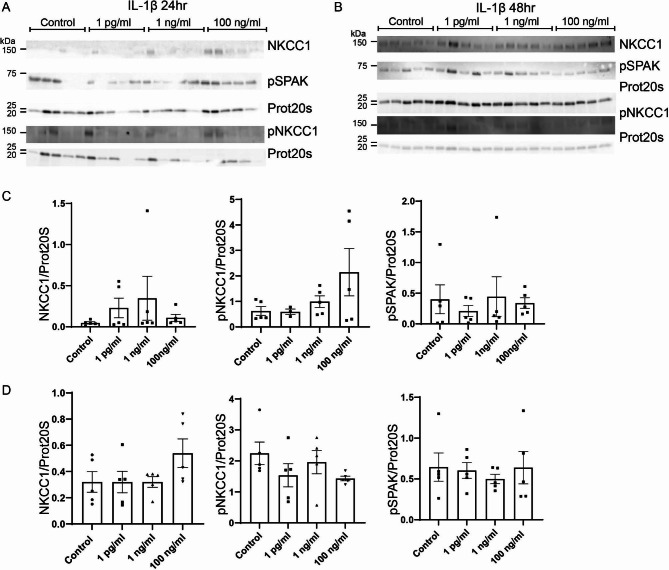
**Effect of the proinflammatory cytokine IL-1β on the CP expression of NKCC1, pNKCC1 and pSPAK.** Primary cultures were treated with either 1 pg/mL, 1 ng/mL, or 100 ng/mL IL-1β for 24 (**A**) or 48 (**B**) hours. Cells were lysed and subjected to immunoblotting for NKCC1, pNKCC1, pSPAK and proteasome 20 S on the same membrane. Molecular masses (shown in kDa) are indicated on the left. The NKCC1, pNKCC1, pSPAK and proteasome 20 S specific bands were quantified densiometrically. Bargraphs in (**C**) and (**D**) show the ratio of NKCC1/proteasome 20 S (left), pNKCC1/proteasome 20 S (middle), and pSPAK/proteasome 20 S (right) as mean ± SEM.

Treatment with IL-1β did not show the same pattern as TNFα. Treatment with IL-1β for 24 h (Fig. 4A and C) did not affect NKCC1 at either dose (1 pg/mL, 1 ng/mL, pr 100 ng/mL). Unlike TNFα, 24 h treatment with IL-1β had no effect on pNKCC1 abundance. Finally, pSPAK levels were also unaffected by IL-1β treatment. The abundance of each protein remained unchanged after 48 h of treatment with IL-1β (Fig. 4B and D). Total NKCC1 was unaffected by 1 pg/mL, 1 ng/mL and 100 ng/mL IL-1β. The effect on pNKCC1 remained absent after 48 h of treatment. Finally, pSPAK abundance was similarly unaffected by treatment after 48 h.

### Acute treatment of the CPE cultures with IL-6 transiently increases abundance of pSPAK and pNKCC1

**Fig. 5:**
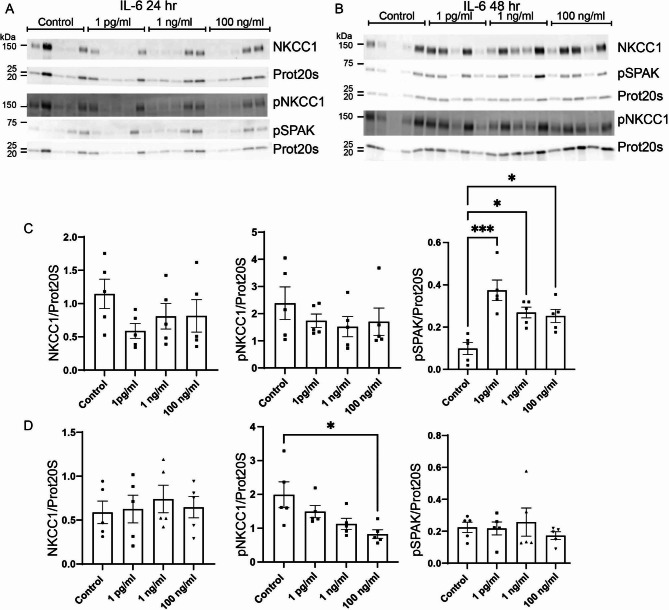
**Effect of the proinflammatory cytokine IL-6 on the CP expression of NKCC1, pNKCC1 and pSPAK.** Primary cultures were treated with either 1 pg/mL, 1 ng/mL, or 100 ng/mL IL-6 for 24 (**A**) or 48 (**B**) hours. Cells were lysed and subjected to immunoblotting for NKCC1, pNKCC1, pSPAK and proteasome 20 S on the same membrane. Molecular masses (shown in kDa) are indicated on the left. The NKCC1, pNKCC1, pSPAK and proteasome 20 S specific bands were quantified densiometrically. Bargraphs in (**C**) and (**D**) show the ratio of NKCC1/proteasome 20 S (left), pNKCC1/proteasome 20 S (middle), and pSPAK/proteasome 20 S (right) as mean ± SEM. *indicates p < 0.05 and ***p < 0.001

IL-6 activates the JAK/STAT pathway through the IL-6 receptor located in the basolateral membrane of the CP. Treating the cells for 24 h with IL-6 (Fig. 5A and C) did not affect the abundance of total NKCC1 or pNKCC1 but increased abundance of pSPAK. NKCC1 was unaffected by treatment with either 1 pg/mL, 1 ng/mL, or 100 ng/mL IL-6. Similarly, the activated form of NKCC1, pNKCC1 was not affected by IL-6 treatment by either 1 pg/mL, 1 ng/mL, and 100 ng/mL. The abundance of the activator of NKCC1, pSPAK, was, however, increased by 74% after 24 h treatment with 1 pg/mL IL-6 (95% CI of diff. [-0.41 to -0.14], p = 0.0002), by 63% with 1 ng/mL (95% CI of diff. [-0.31 to -0.03], p = 0.0149) and by 61% with 100 ng/mL (95% CI of diff. [-0.29 to -0.01], p = 0.0288).

Treatment for 48 h with IL-6 (Fig. 5B and D) had no effect on total NKCC1. IL-6 did, however, affect the phosphorylated form of NKCC1 after 48 h. Despite the activation of pSPAK at 24 h, pNKCC1 abundance after 48 h was reduced by 58% by the highest dose of IL-6 only (100 ng/mL: 95% CI of diff. [0.21 to 2.11], p = 0.0145). The lower doses did not affect pNKCC1. The effect on pSPAK observed after 24 h treatment was transient and pSPAK was normalized after 48 h of IL-6 treatment.

### NKCC1 is transiently activated by IL-6 treatment but decreased by TNFα and IL-1β

**Fig. 6:**
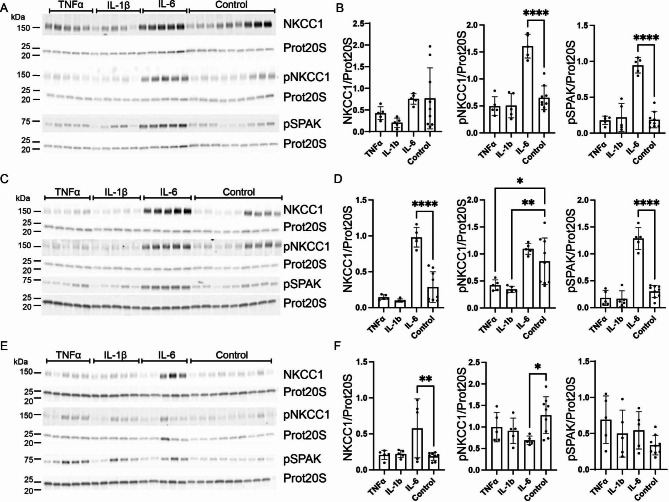
**Effect of TNFα, IL-1β and IL-6 on CP expression of NKCC1, pNKCC1 and pSPAK.** Primary culture cells were treated with either 100 ng/mL TNFα, IL-1β, or IL-6 for 24 h (**A** + **B**), 48 h (**C** + **D**) or 72 h (**E** + **F**). Cells were lysed and subjected to immunoblotting for NKCC1, pNKCC1 and pSPAK as well as proteasome 20 S (Prot20S) on the same membrane. Molecular masses (shown in kDa) are indicated on the left. The proteins were detected and densiometrically quantified. Bar graphs show expression of NKCC1 (left), pNKCC1 (middle) and pSPAK (right) relative to proteasome 20 S as mean ± SEM .*indicates p < 0.05, **p < 0.01, and ****p < 0.0001

Similar to the effect observed on Ncbe, the results indicated a transient pattern in activation of NKCC1. The effect of the cytokines was therefore investigated at the highest dose but over a longer period (Fig. 6). In the repeated experiments abundance of total NKCC1 remained unaffected by treatment of the cell cultures with either TNFα or IL-1β after 24 h (Fig. 6A and B), 48 h (Fig. 6C and D), or 72 h (Fig. 6E and F).

The abundance of pNKCC1 was unaffected by both TNFα and IL-1β after 24 h treatment (Fig. 6A and B). This contrasts with the previous experiment that showed a significant decrease in pNKCC1 after 24 h of treatment with TNFα.

Treatment with TNFα and IL-1β for 48 h, however, decreased pNKCC1 abundance by 51% (95% CI of diff. [0.04 to 0.85], p = 0.0289) and 60% (95% CI of diff. [0.12 to 0.92], p = 0.0099), respectively (Fig. 6C and D). The effect of 48 h treatment with TNFα on pNKCC1 is similar to that observed at 24 and 48 h in the previous experiments. The pNKCC1 levels are normalized after 72 h (Fig. 6F) confirming a transient nature of this activation. The reduced abundance of pNKCC1 after 48 h of treatment with IL-1β contrasts with the effect of the same treatment time in the previous experiments. The effect is however also transient as the levels of pNKCC1 normalize after 72 h (Fig. 6E) suggesting a narrow window where the increased abundance can be detected.

Similar to the previous experiments, neither TNFα nor IL-1β treatment affected pSPAK after 24 h (Fig. 6A and B), 48 h (Fig. 6C and D), or after 72 h (Fig. 6E and F).

Like the previous experiments, treatment of the primary CPE cell culture with 100 ng/mL IL-6 did not affect total NKCC1 abundance after 24 h (Fig. 6A and B). NKCC1 abundance was however increased by 242% (95% CI of diff. [-0.91 to -0.48], p < 0.0001, Fig. 6C and D) and 206% (95% CI of diff. [-0.67 to -0.10], p < 0.0069, Fig. 6E and F) after 48 and 72 h, respectively.

Both pNKCC1 and pSPAK increased greatly in abundance after 100 ng/mL IL-6 treatment. The abundance of pNKCC1 increased after 24 h by 147% (95% CI of diff. [-1.26 to -0.65], p < 0.0001, Fig. 6A and B). After 48 h, however, pNKCC1 returned to control culture levels (Fig. 6C and D) and after 72 h pNKCC1 was reduced by 46% (95% CI of diff. [0.10 to 1.07], p = 0.0166, Fig. 6E and F). The increase after 24 h therefore is transient and the timeframe of seeing the abundance increase is most likely narrow. Like in the previous experiments, the abundance of pSPAK increased after 24 h by 406% (95% CI of diff. [-0.94 to -0.58], p < 0.0001, Fig. 6A and B). Unlike pNKCC1, pSPAK levels remained elevated after 48 h by328% compared to control (95% CI of diff. [-1.20 to -0.78], p < 0.0001, Fig. 6C and D). After 72 h, pSPAK normalized to control levels (Fig. 6E and F) again suggesting a transient pattern in activation of NKCC1.

## Discussion

In this study, we have investigated the response of CPE cells to the inflammatory conditions that mimic IVH by studying the individual effects of three dominating proinflammatory cytokines found in CSF of patients following IVH.

We find that two key transporters, NKCC1 and Ncbe, involved in CSF secretion by the CP respond in a different pattern to the same inflammatory stimulus. NKCC1 seems to be stimulated primarily via IL-6 and Ncbe is affected primarily by TNFα and IL-1β.

**Fig. 7:**
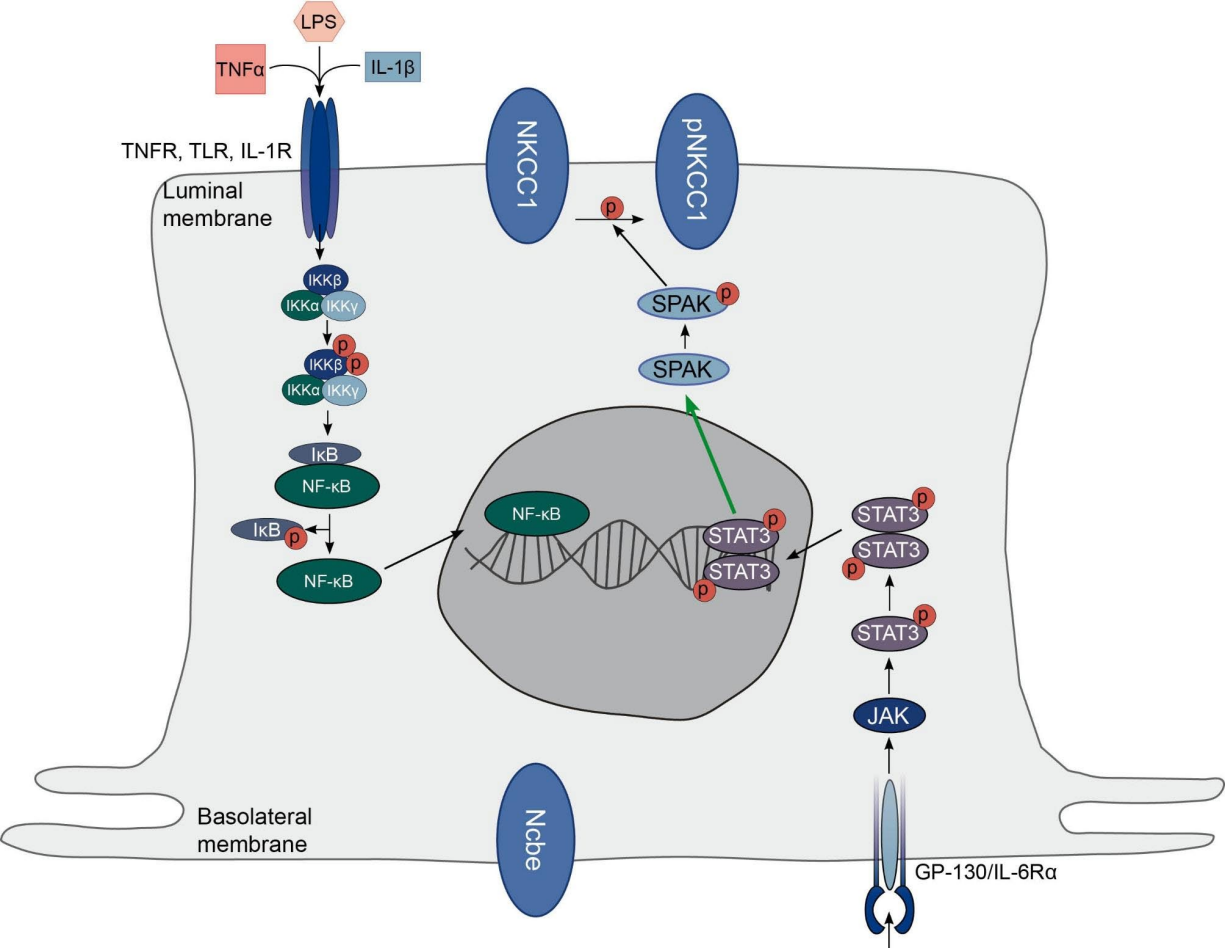
**Schematic summation of the proposed pathway of activation of the luminal NKCC1 in the CP.** The pro-inflammatory cytokines TNFα and IL-1β as well as LPS activate receptors in the luminal membrane of the CP that faces the CSF. This initiates the activation of the NF-κB pathway. The receptor for IL-6 is present in the basolateral membrane and activates the JAK/STAT pathway. In this study, IL-6 leads to increased abundance of pNKCC1 in the luminal membrane, whereas the activation of the luminal receptors has little or no effect on either Ncbe and NKCC1. Please see text for further details

During IVH several cytokines are released into the CSF. The three most prevalent and most abundant cytokines in CSF from SAH patients are the pro-inflammatory cytokines TNFα, IL-1β, and IL-6 [7]. The data is achieved with a bench top assay and are biased towards the cytokines chosen to test. Studies on inflammatory content of SAH patients compared to control subjects (acute and over time) have, however, revealed a range of dysregulated inflammatory markers [30, 33]. TNFα and IL-1β both act on receptors that are located in the luminal membrane of the CPE cells, similar to TLR4 (Fig. 7) [28, 29]. TNFα binds to the TNFR and IL-1β binds to the IL-1 receptor. The TLR4, TNFR, as well as the IL-1 receptor signal through the NFkB pathway. The decreased abundance of the basolateral transporter Ncbe, by both TNFα, and IL-1β that activate a luminal receptor indicates that this transporter in the basolateral membrane is part of the response to a luminal activator found in the CSF (Fig. 7). Whether the effect on Ncbe is a direct effect mediated by the NFkB pathway or is mediated by changes in the gradients of the ions across the epithelium will need further investigation. Previous studies have shown that the CP barrier function may be disrupted by TLR activation in postnatal mice [34].

IL-6 is rapidly and transiently produced in response to tissue damage and infections. It contributes to the defense of the host through acute phase immunological reactions [35]. In the CP, IL-6 is mainly found to be produced by the stromal cells, and acts on IL-6Rα which is predominantly expressed on the basolateral side of the CPE cells [29]. In this study, IL-6 had no effect on Ncbe abundance but led to an increase in both pSPAK and pNKCC1 abundance. Previous studies show that inflammation of the CP following IVH in rat models leads to activation of NKCC1 and pSPAK [6, 30]. In the rat model, the inflammation was shown to be dependent on the presence of TLR4. Our study indicates that the TLR4 is not directly involved in the activation of NKCC1 in mice but rather that NKCC1 is activated by IL-6 in the basolateral membrane. In this study, IL-6 was added to the cell culture media and therefore had access to both membranes. During IVH, IL-6 would have to cross the blood CSF barrier to reach the basolateral membrane. Higher systemic IL-6 levels have indeed been shown in patients following SAH [36]. The two most predominant cytokines found in SAH patients are as mentioned TNFα and IL-1β. In severe cases also IL-6 is present. In the study by Karimy et al. [6], the authors find CSF hypersecretion 24 h after blood is injected into rat brain ventricles and show evidence that the TLR4 receptor is necessary for the activation of the TLR4- NFkB pathway.

In the CP, TLR4 is predominantly located in the luminal CSF facing membrane (Fig. 7). Lipopolysaccharide (LPS) has been used in many studies to induce inflammation. LPS is an endotoxin that activates a cellular response through the Toll like receptor 4 (TLR4) pathway and also initiates the NFkB signaling pathway. The LPS receptor mCD14 has been located in the CP luminal membrane in rat [37, 38] and the Toll-like receptor 4 (TLR4) is expressed in the mouse CP [39, 40]. In our mouse cell culture, the basolateral Na^+^ loader, Ncbe, transiently decreased in response to LPS (data not shown). Except for a transient decrease in abundance in pNKCC1, however, treatment with LPS had no effect on pSPAK, pNKCC1 and NKCC1 abundance (data not shown), further suggesting that there is no direct coupling between TLR4 activation and NKCC1 phosphorylation. Our TEER data, however, could suggest acute disruption of the CP barrier at least with high concentrations of TNFα and IL-1β. The decrease in TEER is, however, transient with TNFα and IL-1β and only after 24 h. The effect of the cytokines on the blood-CSF barrier and the impact on CSF secretion does, however, need to be investigated further, preferably in an in vivo model. As previously mentioned, a major complication from IVH is PHH that, at least to some extent, is mediated by an inflammation-dependent hypersecretion of CSF from the choroid plexus [6] possibly mediated by a “cytokine storm” triggered by macrophages derived both peripherally and associated to the CP [41]. To mediate net CSF secretion, the CP is dependent on net transport of Na^+^ from blood to CSF. If no Na^+^ is transported by the basolateral transporters, the luminal transporters will have to recycle Na^+^ back and forth from the CSF to the epithelial cells. Ncbe is a Na^+^ loader in the basolateral membrane of the CP and thereby potentially also a major component in CSF secretion. Therefore, it is surprising that Ncbe abundance either is unchanged or decreases in these experiments where CSF secretion is expected to increase. The abundance of Ncbe is not equal to activity of the protein, but a decrease in abundance does suggest that basolateral Na^+^ transport mediated by Ncbe is decreased. Further studies are however necessary to investigate if Ncbe in fact does decrease activity during IVH induced inflammation of the CP in vivo.

The present study is based on a primary cell culture model in which the choroid plexus from one mouse pup is seeded onto a filter in one well and each well represents one n. This does create inter-filter variability as can be observed in the TEER measurements. The primary culture was validated and based on a similar rat primary culture previously thoroughly validated [42]. Pooling the choroid plexus from multiple pups and seeding a mixed cohort onto multiple filters might possibly have lessened interfilter-variabilty. The current protocol was however chosen for multiple reasons including difference in littersizes and minimizing risk of infection from the animal facilities. In our opinion, the current setup where one well represents one mouse makes the variability equal to an animal experiment where each animal represents an n.

## Conclusion

Our study highlights the complexity of the pathophysiology behind hypersecretion of CSF because of an IVH that eventually leads to PHH. Our data suggest that the inflammatory pathway involved in hypersecretion primarily occurs as a consequence of activation of basolateral receptors in the CP mediated primarily by IL-6. Future studies will be necessary to investigate the integrity of the barrier following IVH.

## Data Availability

We confirm that all data from this study will be available upon request.

## List of abbreviations

Ncbe: Na^+^ dependent Cl^−^/HCO_3_^−^ exchanger
NKCC1: Na^+^ K^+^ 2Cl^−^ cotransporter 1
CSF: Cerebrospinal fluid
CP: Choroid plexus
CPE: Choroid plexus epithelia
IVH: Intraventricular hemorrhage
TNF: Tumor necrosis factor
IL: Interleukin
LPS: Lipopolysaccharide
ICP: Intracranial pressure
PHH: Posthemorrhagic hydrocephalus
BCSFB: Blood-cerebrospinal-barrier
